# Cost analysis for cancer subgroups

**DOI:** 10.1038/sj.bjc.6605035

**Published:** 2009-04-28

**Authors:** P S Hall, T A Rautenberg, C McCabe

**Affiliations:** 1 Section of Oncology and Clinical Research, Leeds Institute of Molecular Medicine, University of Leeds, Leeds, UK; 2 Academic Unit of Health Economics, Leeds Institute of Health Sciences, University of Leeds, Leeds, UK

**Sir,**

We thank Thomas *et al* (2009) for their thorough collection and presentation of resource use and costs for breast cancer relapse. This represents an excellent summary of the costs of advanced breast cancer to the NHS. Current data of this kind is vital for NICE to make decisions based on robust evidence. Detailed inclusion of community costs make this study one of the most comprehensive cost descriptions for advanced breast cancer in the United Kingdom to date. Publication of cost data for the management of cancer in peer-reviewed journals is an important priority for the future. We would like to communicate a need for caution and clarity of direction in how these data are developed.

The authors identified increasing overall treatment costs as a consequence of the introduction of new and expensive targeted therapies such as trastuzumab (Herceptin). The costs for the one-fifth of patients whose tumours overexpress HER2 were suggested to be higher than for other patients. This is an important issue, as there is now good evidence that the biological subgroups of breast cancer have very different characteristics and prognosis (Perou *et al*, 2000; Dent *et al*, 2008). Global clinical trials of new therapies are now looking at these subgroups separately. This will result in very different treatment pathways with differences in resource use and costs and a consequent influence on cost-effectiveness results.

Thomas *et al* (2009) present their cost analysis with a view to future economic evaluation of new targeted therapies for early breast cancer. In addition to calculating a monthly cost per patient from relapse, it would be useful to consider costs, which arise prior to relapse. For example, certain subgroups may accrue more costs due to side effects from adjuvant therapy before relapse, with a lower monthly cost after relapse compared with other subgroups.

Proximity to death (i.e., life expectancy at cancer relapse) has been identified as a predictor of healthcare expenditure (Shang and Goldman, 2007). It is likely to vary significantly between subgroups and should be reported in relation to costs. It would also be interesting to evaluate the relationship between distribution of costs (community care *vs* hospital) and proximity to death, or death itself. This would give us important information regarding patterns of resource utilization, which could then be used for more insightful service planning.

There is therefore a real need for future cost analyses in breast cancer and other cancers to differentiate between biological subgroups. This need is amplified when considering the cost implications of adjuvant therapies specifically targeting these subgroups. Future studies must be larger, with adequate numbers in each subgroup, or be more specifically focused on a single subgroup to provide adequately powered results. Standards must be adopted at a national level for independently collecting subgroup data as malignant diseases become further divided by gene expression profiling with individualised treatment options. Cost analyses such as Thomas *et al* (2009) have provided, represent a first step in this direction and are invaluable in providing evidence for increasingly difficult decisions.
